# A Novel Combined SLAM Based on RBPF-SLAM and EIF-SLAM for Mobile System Sensing in a Large Scale Environment

**DOI:** 10.3390/s111110197

**Published:** 2011-10-28

**Authors:** Bo He, Shujing Zhang, Tianhong Yan, Tao Zhang, Yan Liang, Hongjin Zhang

**Affiliations:** 1 School of Information Science and Engineering, Ocean University of China, 238 Songling Road, Qingdao 266100, China; E-Mails: sjzhang365@gmail.com (S.Z.); zhangtao_ouc@163.com (T.Z.); liangyan1210@126.com (Y.L.); excellentzhj@163.com (H.Z.); 2 School of Mechanical & Electrical Engineering, China Jiliang University, 258 Xueyuan Street, Xiasha High-Edu Park, Hangzhou 310018, China

**Keywords:** RBPF-SLAM, EIF-SLAM, submap, consistency, computational efficiency

## Abstract

Mobile autonomous systems are very important for marine scientific investigation and military applications. Many algorithms have been studied to deal with the computational efficiency problem required for large scale Simultaneous Localization and Mapping (SLAM) and its related accuracy and consistency. Among these methods, submap-based SLAM is a more effective one. By combining the strength of two popular mapping algorithms, the Rao-Blackwellised particle filter (RBPF) and extended information filter (EIF), this paper presents a Combined SLAM—an efficient submap-based solution to the SLAM problem in a large scale environment. RBPF-SLAM is used to produce local maps, which are periodically fused into an EIF-SLAM algorithm. RBPF-SLAM can avoid linearization of the robot model during operating and provide a robust data association, while EIF-SLAM can improve the whole computational speed, and avoid the tendency of RBPF-SLAM to be over-confident. In order to further improve the computational speed in a real time environment, a binary-tree-based decision-making strategy is introduced. Simulation experiments show that the proposed Combined SLAM algorithm significantly outperforms currently existing algorithms in terms of accuracy and consistency, as well as the computing efficiency. Finally, the Combined SLAM algorithm is experimentally validated in a real environment by using the Victoria Park dataset.

## 1. Introduction

Mobile autonomous system are often required to operate in highly uncertain and dynamic environments, such as a battle zone, with minimal or no external help such as GPS or human intervention. Other applications range from disaster relief in areas such as the 9/11 WTC site, to underwater and planetary exploration. Hence, the development of true autonomy in such systems can have very wide military, societal and scientific impact. In order to be “truly autonomous”, any such autonomous system needs to be capable of accomplishing two sub-tasks: (1) it needs to model its environment, along with its attendant uncertainties, as well as find its own location within the environment, using only the noisy observations from its on-board sensors, and (2) it needs to use the environment model to intelligently plan its actions within the environment such that it accomplishes its objectives in a robust and timely fashion. Problem 1 above is known as the Simultaneous Localization and Mapping (SLAM) problem, while the addition of planning to the problem of SLAM in Problem 2 results in the so-called Simultaneous Planning, Localization and Mapping (SPLAM) problem [1,2]. Thus, the SLAM problem is a fundamental problem in enabling “true autonomy”.

After the first closed formulation was introduced, the SLAM problem has attracted immense attention in the mobile robotics fields [3–6]. SLAM approaches can be roughly classified according to the map representation and estimation algorithm used. Popular methods for representing environment maps include the feature-based approach [7], the grid-based approach [8], and the topological approach [9]. A large variety of estimation techniques has been proposed to address the SLAM problem, including the Extended Kalman Filter (EKF) [10], Rao-Blackwellized Particle Filter (RBPF) [11], Unscented Kalman Filter (UKF) [12,13], EIF [14] and several other techniques that have been applied to estimate the trajectory of robots as well as map the environment. Rapid and exciting progress has been made in solving the SLAM problem together with many compelling implementations of SLAM methods during the past decades. However, enabling real-time SLAM implementation in an increasingly unstructured large-scale environment is still a great challenge. With the goal of addressing this problem, submap-based methods are proposed, simultaneously, improving the computational efficiency of SLAM problem [15].

Some scientists have studied the submap-based solution to SLAM problem in large scale environments, e.g., by mixing RBPF with UKF [16], PF with Gaussian filters [17], EKF with EIF [18], and FastSLAM (*i.e.*, RBPF) with EKF [19] *etc*. In this paper, a novel mixed SLAM method, mixing RBPF with EIF, is presented. Its accuracy and consistency outperform the previous mixed methods significantly, especially the computational efficiency. The major contributions of this work are as follows:

- Based on the theoretical analysis, the Combined SLAM, a novel submap-based solution to SLAM problem, is proposed for mobile system sensing in large scale environments. The proposed algorithm combines the strength of two popular mapping algorithms: RBPF and EIF. As RBPF-SLAM can avoid linearization of the robot model during operation and provide a robust data association, it is used to produce local maps which are periodically fused into an EIF-SLAM algorithm. Specifically, the sparse information matrix together with the recovery of state vector and covariance submatrix of EIF algorithm significantly improves the whole computational efficiency, and simultaneously avoids the tendency of RBPF-SLAM to be over-confident.
- In contrast with sequential submap joining strategy, a binary-tree-based decision-making strategy is proposed to merge the submaps efficiently in order to reduce the computational cost and improve the real-time performance. It has been illustrated that the strategy will result in a total computation cost that is less than *O*(*n*log*n*).

The remainder of this paper is structured as follows: after an overview of related work in the next section, the overall structure of the proposed Combined SLAM algorithm is outlined in Section 3. Section 4 describes the probability distribution of RBPF-SLAM algorithm and its conversion, while in Section 5 we provide a brief review of the EIF-SLAM algorithm for fusing local submaps into a global map. Section 6 presents the binary-tree-based decision-making strategy, as well as its computational cost. In Section 7 we carry out a set of experimental comparisons in terms of accuracy and consistency by using simulated and the Victoria Park datasets, while the results are also discussed. Finally, Section 8 summarizes the most important conclusions of this work.

## 2. Related Works

Considering the estimation algorithm for SLAM problem, the most popular one is EKF. The effectiveness of the EKF approach comes from the fact that it estimated a fully correlated posterior over feature maps and robot poses. However, the EKF-based SLAM algorithm suffers from three well-known drawbacks which complicate its application to large real-world environments: quadratic complexity with respect to the size of the map; inconsistency due to its linearization approximation and sensitivity to failures in data association. Many approaches have been developed to overcome these shortcomings, and the most common ones are: (a) RBPF-SLAM, first proposed by Murphy [11], is an effective approach which describes the vehicle motion model as a set of samples of a more general non-Gaussian probability distribution. The RBPF-SLAM algorithm overcomes the linearization problem and is also more robust in data association. Based on the RBPF framework, FastSLAM [20] uses particle filtering to address non-linearity and factorization to avoid large state vectors; (b) EIF-SLAM, an alternative newer method, has been used as a recursion for the inverse of the covariance matrix which has been shown to be exactly sparse with no generation of inaccuracies through sparsification, but abandoning the odometry information.

In order to improve the computational efficiency of SLAM, with the goal of being able to map large scale environments in real time, the global map is decomposed into smaller submaps in the popular algorithms, and then submap joining is implemented to form a global map [21]. The notion has been studied successfully in some references, e.g., The Constrained Local Submap Filter (CLSF) [22], the Hierarchical SLAM [23] or the recent D&C SLAM [24], *etc*., are conducted in realistic environments. Given a map of n features, the classical EKF SLAM algorithm is known to have a cost of O(n^2^) per step. Two recent algorithms have provided important reductions in computational cost: with an amortized cost of O(n) per step, SLSJF SLAM [25] reports a cost O(n^1.5^) per step in the worst cases. The Treemap algorithm [26] has a cost of O(logn), although with topological restrictions on the environment, and a rather complex implementation.

The aforementioned works have solved the large scale SLAM problem in a number of different forms. In this paper, we present a novel combined SLAM: a submap-based approach to SLAM problem which combines the strengths and avoids the weaknesses of two popular mapping strategies: RBPF-SLAM and EIF-SLAM. Local map building is carried out by using the RBPF-SLAM algorithm which does not suffer from linearization problems and is much more robust in association ambiguity situations. After a sequence of submaps is built, each of them is assembled in a single multi-dimensional Gaussian fashion for the subsequent process of submap joining from the RBPF-SLAM results. Then, submap joining is implemented for fusing the built local submaps into a global map by using the EIF-SLAM algorithm, which not only has the advantages of EKF-SLAM, but also decreases the computational complexity and improves the consistency of estimation.

## 3. The Overall Structure of Combined SLAM

**Figure 1.**
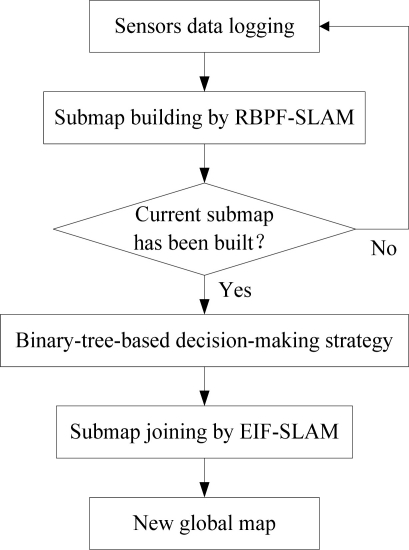
The overall structure of the Combined SLAM algorithm.

The submap-based SLAM problem is how to slice the whole running time of a robot into consecutive periods, and each period has its own beginning. Suppose a local map is expressed by (*X^L^*, *Q^L^*), where *X^L^* (the superscript ‘*L*’ here stands for the local submap) is an estimation of the state vector and *Q^L^* is the associated covariance matrix. State vector *X^L^* contains the final robot pose $X_{s}^{L}={\left(x,\;y,\;\varphi \right)}^{T}$ which includes the robot position *x*, *y* as well as its orientation *φ* and all the local feature positions which are shown as $X_{1}^{L},\;\ldots ,\;X_{n}^{L}$. The coordinate frame of a local submap is defined by the first robot pose when building of this submap started. It is assumed that in real applications the robot will start to build the (*k* + 1)th local map as soon as local map *k*th is finished, thus the robot ending pose in the *k*th local map is the same as the robot starting pose in the (*k* + 1)th local map. The configuration of the proposed Combined SLAM algorithm is outlined in the following flow chart (Figure 1). More details about the highlights will be given in the following sections.

## 4. RBPF-SLAM Algorithm: Probability Distributions and Its Conversion

In this section, we will address the Rao-Blackwellized particle filter used in submap building and particularly discuss the probability distribution of RBPF-SLAM and its conversion.

### 4.1. Probabilistic SLAM

In probabilistic form, the SLAM algorithm can be described by [3]:

(1) $$p\left(S^{t},\;M|Z^{t},\;C^{t},\;A^{t}\right)$$

where *S^t^* = *S*_1_, *S*_2_, …, *S_t_* stands for the path of robot; *M* = *θ*_1_, *θ*_2_, …, *θ_n_* denotes the positions of a set of features which have been identified in the map; *Z^t^* represents the set of all observations; *C^t^* refers to the set of controls; *A^t^* denotes the data associations. The subscript *t* is used to indicate the variable at each time-step *t*, while the superscript *t* indicates the set of variables for all time-steps up to *t* and including time-step *t*. Marginalizing out the past poses, SLAM algorithms only estimate:

(2) $$p\left(S_{t},\;M|Z^{t},\;C^{t},\;A^{t}\right)$$

When a submap is fusing into the global map, EIF-SLAM algorithm requires this submap be subject to the above distribution in the form of a single multi-dimensional Gaussian. However, the probability distribution of RBPF-SLAM algorithm is a Gaussian Mixture Model (GMM; it will be presented in Section 4.2). Therefore, we should convert the Gaussian Mixture Model of RBPF-SLAM into a single multi-dimensional Gaussian model for the usage of EIF-SLAM.

### 4.2. Gaussian Mixture Model (GMM) of RBPF-SLAM

In the RBPF-SLAM algorithm, Equation (1) can be factored as follows [20]:

(3) $$p\left(S^{t},\;M|Z^{t},\;C^{t},\;A^{t}\right)=p\left(S^{t}|Z^{t},\;C^{t},\;A^{t}\right)\prod_{i=1}^{n}\;p\left(\theta_{i}|S^{t},\;Z^{t},\;C^{t},\;A^{t}\right)$$

where the first factor represents the robot pose and the subsequent factors indicate feature positions given the robot pose. Generally, this factored distribution is represented as a set of *K* particles, with the *j*th particle $P_{t}^{j}$ consisting of an importance weight $w_{t}^{j}$, a robot pose $S_{t}^{j}$, and *n* Gaussian feature estimations described by their mean $\mu_{t}^{j}$ and covariance $\Sigma_{n,t}^{j}$, and the form is as follows:

(4) $$P_{t}^{j}=<w_{i}^{j},\;S_{t}^{j},\;\mu_{1,t}^{j},\;\Sigma_{1,t}^{j},\ldots ,\mu_{n,t}^{j},\;\Sigma_{n,t}^{j}>$$

In order to represent the distribution of each particle as low-dimensional Gaussians, the particle can equivalently be represented as:

(5) $$P_{t}^{j}=<w_{t}^{j},\;x_{t}^{j},\;Q_{t}^{j}\;>$$

where $x_{t}^{j}=\left[S_{t}^{j},\;\mu_{1,t}^{j},\;\ldots ,\;\mu_{n,t}^{j}\right]$ denotes the concatenation of the robot poses with all feature states, and $Q_{t}^{j}$ denotes a block-diagonal covariance matrix which is constructed from the robot covariance and the covariance of each feature, which is shown as:

(6) $$Q_{t}^{j}=\left[\begin{array}{cccc} Q_{\mathrm{SS},t}^{j} & & & \\ & \Sigma_{1,t}^{j} & & \\ & & \ddots & \\ & & & \Sigma_{n,t}^{j} \end{array}\right]$$

where $Q_{\mathrm{SS},t}^{j}$ denotes the robot covariance and it is zero because each particle has no uncertainty associated with the robot pose.

### 4.3. Conversion to a Single Multi-Dimensional Gaussian

A single multi-dimensional Gaussian model with mean *x_t_* and covariance *Q_t_* can be obtained from the GMM by using a process known as moment matching [27]:

(7) $$x_{t}=\sum_{K}w_{t}^{j}\;x_{t}^{j}$$

(8) $$Q_{t}=\sum_{K}w_{t}^{j}\;\left[Q_{t}^{j}+\left(x_{t}^{j}-x_{t}\right){\left(x_{t}^{j}-x_{t}\right)}^{T}\right]$$

The first term in the square bracket of Equation (8) denotes the covariance of individual particle which is caused by sensor noise, while the second term shows the variation between particles caused by robot noise.

In the real implementation, every particle of RBPF-SLAM has its own data association decisions, consequently, both the number of features and their ordering may differ between particles. In order to convert the Gaussian Mixture Model (GMM) of RBPF-SLAM into a single multi-dimensional Gaussian model for the usage of EIF, *i.e.*, carry out Equations (7) and (8) successfully, it is necessary to track the correspondences between features of each particle. To achieve this conveniently, related processes are ordered as the following:

1. Assign a unique index to each observation. The form of each particle can be augmented with a set of extra correspondence variables $\lambda_{i,t}^{j}$, then $\lambda_{i,t}^{j}=\Theta$ represents that the *i*th feature in the *j*th particle at time step *t* corresponds to the Θth observation in the environment. Therefore the form of each particle can be reformed as:
  (9) $$P_{t}^{j}=<w_{t}^{j},\;S_{t}^{j},\;\mu_{1,t}^{j},\;\Sigma_{1,t}^{j},\;\lambda_{1,t}^{j},\ldots ,\mu_{n,t}^{j},\;\Sigma_{n,t}^{j},\;\lambda_{n,t}^{j}>$$
2. Apply the maximum likelihood data association in every particle. After the process of data association, we can use its results to implement particle updating and particle augmenting for each particle showed in Equation (9).
3. Re-arrange and re-modify each particle according to a standard which is decided by the particle with the highest importance weight, so as to produce a common features set. The process is accomplished as follows:

Let *δ* denote the reverse function of $\lambda_{i,t}^{j}$, and equation *δ_t_*(Θ, *j*) = *i* indicates that the Θth observation in the common feature set corresponds to the *i*th feature in the *j*th particle. Given these variables, the mean in Equation (5) can be represented as:

(10) $$x_{t}^{j}=\left[S_{t}^{j},\;\mu_{\delta_{t}(1,j),t}^{j},\ldots ,\mu_{\delta_{t}(n,j),t}^{j}\right]$$

and its related covariance is:

(11) $$Q_{t}^{j}=\left[\begin{array}{cccc} Q_{\mathrm{SS},t}^{i} & & & \\ & \Sigma_{\delta_{t}(1,j),t}^{j} & & \\ & & \ddots & \\ & & & \Sigma_{\delta_{t}(n,j),t}^{j} \end{array}\right]$$

In such case that a common feature may have no corresponding feature in any particle, then the particle will be discarded. Through the above three steps, each particle obtains a single multi-dimensional mean and covariance, and a single Gaussian distribution can then be produced by using Equations (7) and (8).

## 5. Submap Joining Procedure

After submaps are established, the submap joining will be implemented. In this section, we will describe the submap joining algorithm based on an extended information filter.

### 5.1. Problem Description

In the local submap joining, the input is an estimation only related to “local” information. If the features among the sequent local submaps are not dense, then the sparsification can be implemented to achieve the map joining effectively. The most important thing is that when the distance among the submaps becomes farther, the correlation between the features of the submaps will be lower. This will result in an exactly sparse information matrix without any approximation, so the EIF-SLAM algorithm will maintain lower computational complexity than EKFSLAM. The state vector of the global map in the proposed algorithm contains feature positions and robot ending poses of each local submap. As usual, the starting point of the first local map is also taken as the starting point of the global map. After the *k*th local map is built, it will be fused into the global map. Suppose the current global state vector is given as *X^G^*(*k*) (the superscript ‘*G*’ here stands for the global map), it can be expressed as follows:

(12) $$\begin{array}{ll} X^{G}(k)= & (X_{1}^{G},\cdots ,X_{n1}^{G},\;X_{1,\;e}^{G},\\ & X_{n1+1}^{G},\cdots ,X_{n1+n2}^{G},\;X_{2,\;e}^{G},\\ & \cdots \\ & X_{n1+\ldots +nk-1+1}^{G},\cdots ,\;X_{n1+\cdots +nk-1+nk}^{G},\;X_{k,\;e}^{G}) \end{array}$$

where $X_{1}^{G},\;\ldots ,\;X_{n1}^{G},\;X_{1,e}^{G}$ are the global positions of environment features and the robot ending global pose $\left(x_{1,e}^{G},\;y_{1,e}^{G},\;{ø}_{1,e}^{G}\right)$ of the 1st local map which is also the starting global position of the 2nd local map. Correspondingly, $X_{n1+1}^{G},\;\ldots ,\;X_{n1+n2}^{G},\;X_{2,e}^{G}$, are the global positions of those detected features in the 2nd local map but not in the 1st local map, together with the robot ending pose. Here the subscript ‘*e*’ stands for ‘ending pose’. Instead of the global state vector estimation *X^G^*(*k*) and its associated covariance matrix *Q^G^*(*k*) used in EKFSLAM, an information vector *i*(*k*) and an associated information matrix *I*(*k*) are defined to express the Gaussian distribution in EIFSLAM, because EIF is an algebraic equivalent to the EKF. The relationship between them can be denoted by:

(13) $$I(k)\;X^{G}\;(k)=i(k),\;\;Q^{G}\;(k)=I{\left(k\right)}^{-1}$$

When the Extended Information Filter is applied to the estimation problem, an off-diagonal element of the information matrix is non-zero only when the two related objects (the features and the robot starting/ending poses) are within the same local map. Since the size of each local map is limited, any objects will only link to their nearby objects, no matter how many (overlapping) local maps are fused. This results in an exactly sparse information matrix without any approximation. Since all the objects involved in the local maps are included in the global state vector, no marginalization is required in the map joining process and thus the information matrix *I*(*k*) will stay exactly sparse all the time and can be computed efficiently. Then *X^G^*(*k*) can be recovered by Equation (13). Even for data association, only a small part of information matrix is handled, so the whole dense covariance matrix doesn’t need to be recovered. After the (*k* + 1)th local map is built completely, features that have not been included in the global map of this local map yet together with the robot ending pose of this local map are fused into the global map, so the new state vector becomes:

(14) $$\begin{array}{ll} X^{G}\;(k)= & (X_{1}^{G},\cdots ,\;X_{n1}^{G},\;X_{1,\;e}^{G},\\ & X_{n1+1}^{G},\;\cdots ,\;X_{n1+n2}^{G},\;X_{2,\;e}^{G},\\ & \ldots \\ & X_{n1+\cdots +nk-1+1}^{G},\cdots ,\;X_{n1+\cdots nk-1+\mathrm{nk}}^{G},\;X_{k,\;e}^{G}\\ & X_{n1+\cdots +\mathrm{nk}+1}^{G},\cdots ,\;X_{n1+\cdots \mathrm{nk}+\mathrm{nk}+1}^{G},\;X_{k+1,\;e}^{G}) \end{array}$$

### 5.2. Implementation of Joining

When the (*k* + 1)th local submap is built, it will be implemented by the four following steps to produce a new global map.

#### 5.2.1. Data Association

Data association aims to find the features in the (*k* + 1)th local map that already exist in the global map. This is a necessary step in SLAM problems using practical data. First, the proposed algorithm determines a set of potentially overlapping local maps corresponding to the (*k* + 1)th local map, and then finds the set of potentially matched features in the potentially overlapping local maps by using simple Euclidean distance. Second, the covariance submatrix associated with the pose and the potentially matched features is recovered based on [28]. In order to improve the computational speed and keep the information matrix positive and definite, Cholesky factorization is sometimes applied to solve Equation (13), and the factoring process is needed to satisfy the equation *F*(*k*)*F*(*k*)*^T^* = *I*(*k*). At last, all the feature positions need to be transferred into the same coordinate system to implement the simple Nearest Neighbor (NN) method for realizing the final match. Algorithm 1 is listed to illustrate the process.

**Algorithm 1.** Algorithm of data association between the global map and the (*k* + 1)th local map.

Input: the (*k* + 1)th local map and global map
(1) Find a set of potentially overlapping local maps
(i) Compute the distance *D_i_* which is from the starting position of the (*k* + 1)th local map to the starting position of the (*k* + 1)th local map (0 ≤ *i* ≤ *k*);
(ii) Set a threshold TH1 (usually the range of the detection sensor);
(iii) If *D_i_* ≤ 2 × TH1, the *i*th local map is a potential candidate.
(2) Find the set of potentially matched features
(i) Set another threshold TH2;
(ii) Find the features of the potential local maps whose Euclidean distance are smaller than the TH2;
(3) Recover the covariance submatrix associated with the pose and the potentially matched features;
(4) Use NN method to identify the final match.

#### 5.2.2. Initialize the New Features in Current Global Coordinate System

After data association, the new features as well as the ending pose in the (*k* + 1)th local map are initialized into the global map. As a result, a new state vector *X^G^*(*k*) is formed. Zeros are added respectively into the information vector *i*(*k*), information matrix *I*(*k*) and its Choleshy factorization *F*(*k*) to produce corresponding vector/matrix.

#### 5.2.3. Update the Global Map

Suppose we have got the (*k* + 1)th local map that is given by Equation (12). Then the submap joining based on EIF-SLAM involves treating the total information of the (*k* + 1)th local map as an observation with a zero-mean Gaussian observation noise whose covariance matrix is *Q^L^*. In this paper, after data association, we suppose the relationship between the features in the (*k* + 1)th local map and the ones in the global map are denoted as $X_{1}^{L}\;↔\;X_{k+1,1}^{G},\;\ldots ,\;X_{n}^{L}\;↔\;X_{k+1,n}^{G}$. And the state estimation in the (*k* + 1)th local map ${\hat{X}}_{k+1}^{L}$ can be regarded as an observation from robot starting pose $X_{k,e}^{G}$ to robot ending pose $X_{k+1,e}^{G}$ and the features $X_{k+1,1}^{G},\;\ldots ,\;X_{k+1,n}^{G}$, which can be represented by the following observation equation:

(15) $$Z={\hat{X}}_{k+1}{}^{L}=H_{k+1}(X^{G})+w_{k+1}$$

where *H*_*k*+1_(*X^G^*) is given by:

$$\left(\begin{array}{c} \left(x_{k+1,e}^{G}-x_{k,\;e}^{G}\right)\text{cos}\varphi_{k,\;e}^{G}+\left(y_{k+1,\;e}^{G}-y_{k,\;e}^{G}\right)\;\text{sin}\;\varphi_{k,\;e}^{G}\\ \left(y_{k+1,\;e}^{G}-y_{k,\;e}^{G}\right)\;\text{cos}\;\varphi_{k,\;e}^{G}-\left(x_{k+1,e}^{G}-x_{k,\;e}^{G}\right)\;\text{sin}\;\varphi_{k,\;e}^{G}\\ \varphi_{k+1,S}^{G}-\varphi_{k,S}^{G}\\ \left(x_{k+1,1}^{G}-x_{k,\;e}^{G}\right)\;\text{cos}\;\varphi_{k,\;e}^{G}+\left(y_{k+1,1}^{G}-y_{k,\;e}^{G}\right)\;\text{sin}\;\varphi_{k,\;e}^{G}\\ \left(y_{k+1,1}^{G}-y_{k,\;e}^{G}\right)\;\text{cos}\;\varphi_{k,\;e}^{G}-\left(x_{k+1,1}^{G}-x_{k,\;e}^{G}\right)\;\text{sin}\;\varphi_{k,\;e}^{G}\\ \vdots \\ \left(x_{k+1,\;n}^{G}-x_{k,\;e}^{G}\right)\;\text{cos}\;\varphi_{k,\;e}^{G}+\left(y_{k+1,\;n}^{G}-y_{k,\;e}^{G}\right)\;\text{sin}\;\varphi_{k,\;e}^{G}\\ \left(y_{k+1,\;n}^{G}-y_{k,\;e}^{G}\right)\;\text{cos}\;\varphi_{k,\;e}^{G}-\left(x_{k+1,\;n}^{G}-x_{k,\;e}^{G}\right)\;\text{sin}\;\varphi_{k,\;e}^{G} \end{array}\right)$$

and *w*_*k*+1_ is the zero-mean Gaussian “observation noise” with covariance matrix *Q^L^*. Subsequently, Equation (15) is used to update the information vector and the information matrix:

(16) $$\begin{array}{c} I(k+1)=I(k)+\nabla H^{T}\;{\left(Q^{L}\right)}^{-1}\nabla H\\ i(k+1)=i(k)+\nabla H^{T}\;{\left(Q^{L}\right)}^{-1}\left[Z-H\left(X^{G}(k)\right)+\nabla HX^{G}(k)\right] \end{array}$$

where ∇*H* is Jacobian of the transportation matrix *H* with respect to *X^G^*(*k*).

#### 5.2.4. State Vector Recovery

What we need is not the information vector *i^G^*(*k* + 1), but rather the global state estimation *X^G^*(*k* + 1), so Equation (12) is used to recover the *X^G^*(*k* + 1).

## 6. Binary-Tree-Based Decision-Making Strategy

During the global map building, in order to reduce the computational cost and improve the real-time performance, we use a binary-tree-based decision-making strategy to merge the submaps efficiently. In a sense, successful submap building is the first step of successful submap joining. Thus the decision-making strategy will be introduced firstly; it is used to generate a sequence of independent submaps with unequal size, then the built submaps are joined to produce the final global map according to the binary-tree-based strategy.

### 6.1. Decision-Making Strategy of Submap Building

Before submap joining, we should ensure the effectiveness of submap building, which depends not only on the submap building algorithm itself (RBPF-SLAM), but also whether each submap is completed properly. Otherwise, the accuracy of the built submap building will be remarkably reduced due to some problems such as error accumulation and particle depletion, thus affecting the accuracy of the final global map produced by submap joining. Therefore, in decision-making strategy of submap building, several factors should be considered:

The first concern is the situation when there is no feature to be detected over a long period of time. Since the accumulated errors cannot be eliminated while the robot is running, we must reduce the running time of the current submap as far as possible. In such case, a constant threshold time TIME is set to $\text{TIME}=\text{max}\left\{\sigma x/\bar{\sigma v_{x,}},\;\sigma y/\bar{\sigma v_{y,}},\;\sigma \theta /\bar{\sigma w}\right\}$ according to the pose optimal estimation, where *σx*, *σy*, *σθ* are the standard deviation of robot pose and $\bar{\sigma v_{x}}$, $\bar{\sigma v_{y}}$, $\bar{\sigma w}$ are the mean measurement noise of associated sensors.

Secondly, particle diversity must be considered. Within the threshold time, along with the appearing of features, if the diversity problem occurs, the robot should stop the construction of the current submap and start to build a new one.

**Figure 2.**
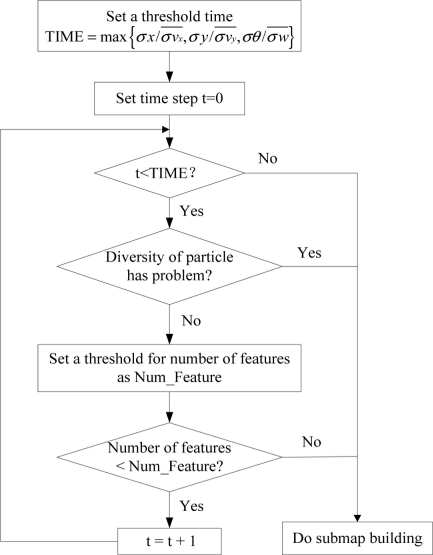
Decision-making Strategy.

The last factor we must consider is the number of features in each submap. Generally, a good rule of thumb is that the ratio of feature number to robot pose dimension should be kept between 5 and 20. If feature number of the selected standard particle used in Section 4.2 arrives at the threshold Num_Feature, then we should cease the construction of current submap immediately. The decision-making strategy is illustrated in the following flow chart (Figure 2):

### 6.2. Binary-Tree-Based Strategy of Submap Joining

**Figure 3.**
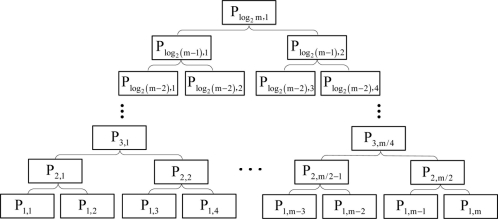
Hierarchical binary-tree-based strategy.

Every built submap should be fused into the global map with a binary-tree-based strategy. The leaves of the binary tree stand for the sequence of the *m* local maps of size *p*_1,*j*_. These maps are joined pairwise to produce *m*/2 local maps with size *p*_2,*j*_, which will be joined pair wise into *m*/4 local maps of size *p*_3,*j*_ in turn, until finally two local maps of size *p*_log_2_*m*−1,*j*_ will be fused into one map, which is the final global map. The hierarchical binary tree fashion is depicted in Figure 3.

The total computational complexity of CombinedSLAM with the binary-tree-based decision-making strategy is:

(17) $$\begin{array}{ll} C & =O\left(\sum_{i=1}^{m}{\mathrm{par}}_{\mathrm{num}}\cdot {\text{log}}_{2}\;p_{1i}+\underset{{\text{log}}_{2}\;m}{\underset{︸}{\sum_{i=1}^{m}p_{1i}+\sum_{i=1}^{\frac{m}{2}}p_{2i}+\sum_{i=1}^{\frac{m}{2^{2}}}p_{3i}+\ldots }}\right)\\ & =O\left({\mathrm{par}}_{\mathrm{num}}\cdot {\text{log}}_{2}\;\prod_{i=1}^{m}\;p_{1i}+\underset{{\text{log}}_{2}m}{\underset{︸}{\sum_{i=1}^{m}p_{1i}+\sum_{i=1}^{\frac{m}{2}}p_{2i}+\sum_{i=1}^{\frac{m}{2^{2}}}p_{3i}+\ldots }}\right)\\ & <O\left({\mathrm{par}}_{\mathrm{num}}\cdot {\text{log}}_{2}{\left(\sum_{i=1}^{m}\;p_{1i}\right)}^{2}+n\;{\text{log}}_{2}\;m\right)\\ & =O\left(2\cdot {\mathrm{par}}_{\mathrm{num}}{\text{log}}_{2}\;n+n\;{\text{log}}_{2}\;m\right)\\ & <O\left(n\;{\text{log}}_{2}\;n\right) \end{array}$$

where *par_num_* is the number of the particles in the RBPF-SLAM algorithm, *m* presents the number of local maps, *n* is the total number of the features, and *p_ij_* denotes the number of the *j*th submap of the *i*th level in the binary tree. The Combined SLAM algorithm offers a reduction in total computational cost that is less than *O*(*n* log_2_ *n*).

## 7. Experiments and Analysis

It can be proven that the EIF is equivalent to the EKF in algebra. EIF has almost the same performance with the EKF except for improving the computational efficiency. Therefore, the following four algorithms in terms of their accuracy, consistency will be compared:

1. The conventional single-map-type EKF-SLAM(EKF);
2. The conventional single-map-type RBPF-SLAM(RBPF);
3. The submap-based SLAM algorithm which combines EKF and EIF(EKF-EIF);
4. The proposed submap-based Combined SLAM: combining RBPF and EIF (RBPF- EIF);

For the computational efficiency, the proposed RBPF- EIF algorithm will be compared with the submap-based SLAM algorithm which combines RBPF and EKF (RBPF-EKF). In order to verify the efficiency of the binary-tree-based decision-making strategy, the comparison will be made also between the conventional single-map-type EKF-SLAM (EKF-SLAM), sequential submap-type Combined SLAM (SS-CombinedSLAM) and binary-tree-based submap-type Combined SLAM (BS-Combined SLAM, *i.e.*, RBPF-EIF).

### 7.1. Experimental Environments of Simulation

**Figure 4.**
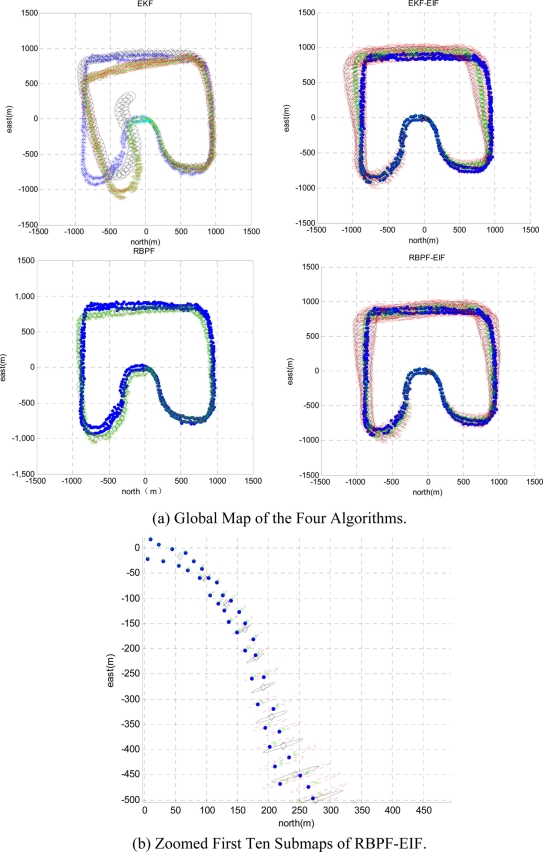
The results of the four algorithms and the zoomed first ten submaps of RBPF-EIF.

The experimental environment consists of 554 randomly-generated point features and simulates a two-dimensional world with a size of 2 km × 2 km, as shown in Figure 4. The robot moves at the speed of 3 m/s with the sampling frequency of 40 Hz, and observes features by using a 180° range-bearing sensor with a maximum range of 30 m and an output frequency of 5 Hz. The standard deviation of robot speed and heading angular are set to 0.3 m/s and 3° respectively. The standard deviations of the observation noise are set to 0.5 m and 5° for range and bearing respectively. The number of the particles is 40 in the particle filter. All algorithms are implemented in Matlab^®^ and executed on 2.60 GHz Pentium^®^ Dual-Core CPU E5300 with 2 GB of RAM.

### 7.2. Experimental Results of Simulation

To compare the performance of the above mentioned four algorithms in a large-scale environment, we use 50 Monte Carlo simulation experiments. The four algorithms run on exactly the same data with a loop closure; data association is known in all algorithms. The results of the four algorithms are shown in Figure 4(a). The left two figures of Figure 4(a) show the map generated by the two conventional sing-map-type SLAM algorithms. One hundred and twenty six small sized submaps are built by the two conventional sing-map-type SLAM algorithms. The right two figures of Figure 4(a) show the global maps generated by fusing all the 126 submaps using the two submap-based SLAM algorithms. In order to compare directly, the estimation results of robot pose and features are displayed with their ground truth respectively. For more clarity, the first ten submaps of RBPF-EIF are zoomed out as shown in Figure 4(b). In Figure 4, the black hollow diamonds indicate the estimation of robot pose, corresponding uncertainty ellipses are given as well. The blue solid dots denote the ground truth positions of features. The green stars denote the estimation positions of features, corresponding uncertainty ellipses are given as well.

#### 7.2.1. Estimation Errors

**Figure 5.**
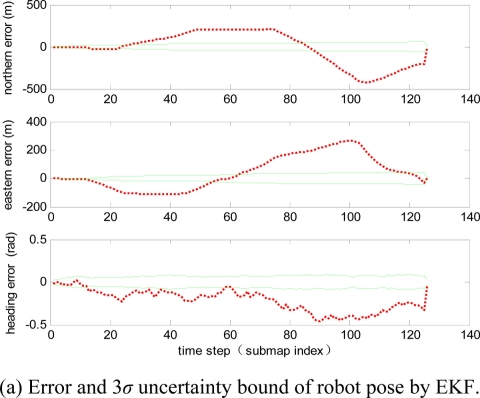

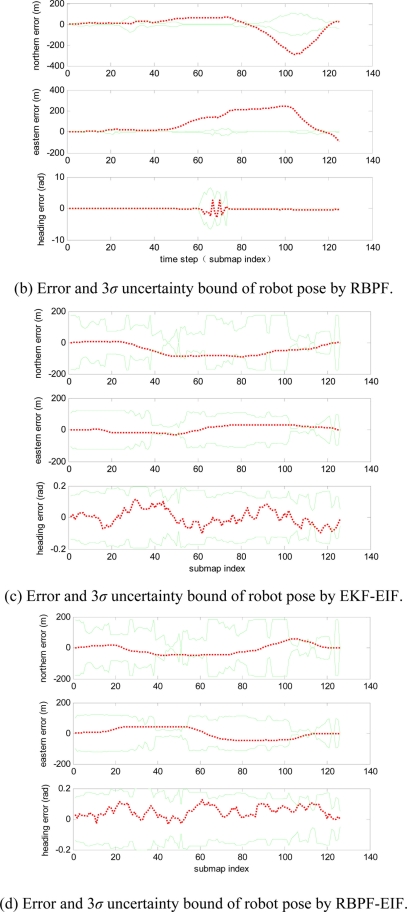
Error and 3*σ* uncertainty bound of robot pose by using the four algorithms, where the red (dot) is errors and the green (solid) is 3*σ* uncertainty bound.

Estimation errors of the four algorithms are shown in Figure 5, where the red dotted line denotes error between the estimation results and the ground truth, and the green solid line shows the 3*σ* uncertainty bound. Obviously, the two conventional single-map-type SLAM algorithms (EKF-SLAM, RBPF-SLAM) will become divergent, as shown in Figure 5(a,b), the other two submap-type SLAM algorithms (EKF-EIF, RBPF-EIF) perform better, as shown in Figure 5(c,d). In the simulation, the robot reduces its localization error and obtains more accurate maps by reviewing old features previously detected in the last submap, and it makes the estimation of submaps perform better.

#### 7.2.2. Consistency

Normalized Estimation Error Square (NEES) and Root Mean Square (RMS) error are used to evaluate the consistency of the proposed filter. When the ground truth *x* for the state vector is available, the NEES could be obtained together with the estimated state vector *x̂* and the covariance matrix P [27]:

(18) $$D^{2}={\left(x-\hat{x}\right)}^{T}P^{-1}\left(x-\hat{x}\right)$$

Consistency is checked up by using the chi-squared criteria:

(19) $$D^{2}<\chi_{n,1-\alpha }^{2}$$

where *n* is the dimension of the variable, and 1 − *α* is the confidence level (95% typically). Then we could define the consistency index of a given estimation (*x̂*, *P*) with respect to its true value *x* as [29]:

(20) $$\mathrm{CI}=\frac{D^{2}}{\chi_{n,1-\alpha }^{2}}$$

When CI < 1, the estimation is consistent with the ground truth, and when CI > 1, the estimation is inconsistent with respect to the ground truth. We tested consistency of the previous four algorithms by carrying out 100 Monte Carlo runs in the simulated experiments.

**Figure 6.**
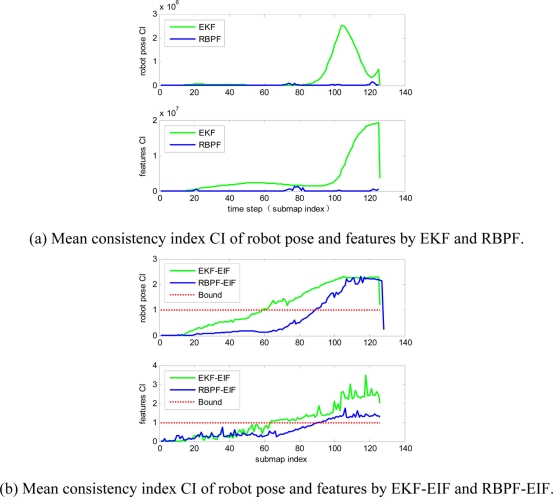
Mean consistency index CI of robot pose and features for the four algorithms.

The mean consistency index CI of robot pose and features for the four algorithms are presented in Figure 6. The two conventional single-map-type SLAM algorithms (EKF-SLAM, RBPF-SLAM) have much bigger CI than the two submap-type SLAM algorithms (EKF-EIF, RBPF-EIF), so they are arranged into two pictures (Figure 6(a,b)), where CI for robot pose and feature are shown in the upper and the lower part of the pictures, respectively. Obviously the estimations of RBPF-SLAM and RBPF-EIF are always more consistent than those of EKF-SLAM and EKF-EIF, and what is more, EKF falls out of consistency at about the 60th submap, while RBPF-SLAM remains consistent.

**Figure 7.**
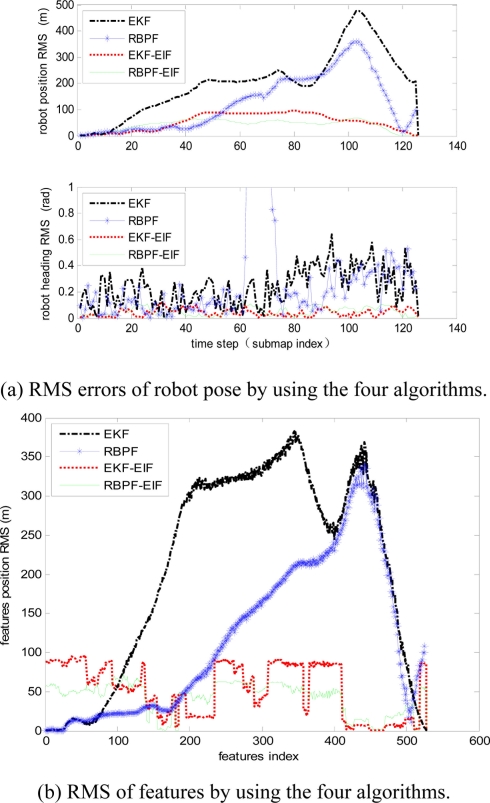
RMS errors of robot pose and feature by using the four algorithms.

The evolution of Root Mean Square (RMS) error on robot position and orientation is shown in Figure 7(a). RMS errors of features by the four algorithms are shown in Figure 7(b). Obviously, RMS errors of submap-based SLAM algorithms are always smaller than the two single-map-based SLAM algorithms. Furthermore, RMS errors of RBPF- EIF SLAM proposed in this paper are better than those of EKF-EIF SLAM.

From parts A and B of the experimental results, it can be noted that among the four algorithms our proposed RBPF-EIF SLAM algorithm demonstrates better performance, especially in dealing with problems as accuracy and consistency in large-scale environment. For the computational cost, the corresponding evaluation is reported as below.

#### 7.2.3. Computational Time

**Figure 8.**
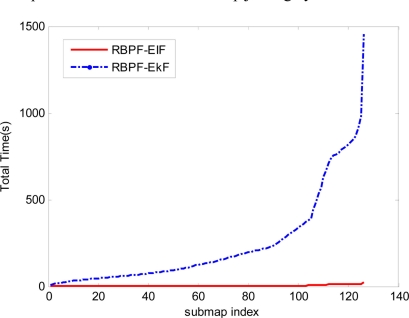
The computational time used for submap joining by RBPF-EKF and RBPF-EIF.

In addition to evaluate accuracy and consistency of the proposed Combined SLAM, we still need to simulate and analyze its computational cost. Table 1 presents the detailed CPU time required for the submap joining by using the RBPF- EKF method and the proposed RBPF- EIF algorithm. The total time for fusing the 126 submaps is about 23 s for EIF and 1,453 s for EKF. It is obvious that the global map updating process takes up most of the computation time in EKF submap joining. Nevertheless, with the sparse information matrix, EIF reduces the updating time to 2.4 s. On the other hand, the major computation time for EIF submap joining is the process of data association which contains the time for covariance matrix recovery. The others including Cholesky factorization and state vector recovery also require a great investment of time. The whole computational time used for fusing the 126 submaps by the classical EKF algorithm and the sparse EIF algorithm is shown in Figure 8.

**Figure 9.**
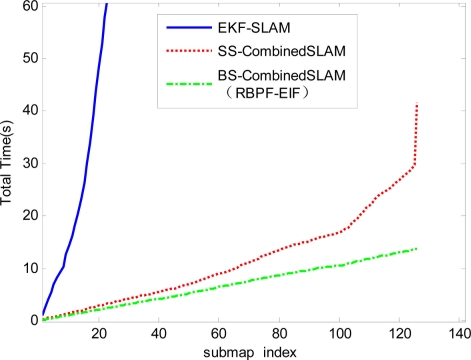
The computation time for three algorithms: conventional single-map-type EKF-SLAM (blue), sequential submap-type Combined SLAM (red) and binary-tree-based submap-type Combined SLAM (green).

In order to validate another advantage of this work, *i.e.*, the binary-tree-based strategy of submap joining, we also choose the conventional single-map-type EKF-SLAM, sequential submap-type Combined SLAM and the binary-tree-based submap-type Combined SLAM for comparison in terms of computation efficiency. The latter two algorithms only differ in the submap joining strategy, and both adopt our proposed Combined SLAM algorithm, using RBPF for submap building and EIF for submap joining as stated before. The computational costs of conventional single-map-type EKF-SLAM (EKF-SLAM), sequential submap-type Combined SLAM (SS-CombinedSLAM) and binary-tree-based submap-type Combined SLAM (BS-CombinedSLAM, *i.e.*, RBPF-EIF) are shown in Figure 9. It can be seen that computational time of the latter two submap-type Combined SLAM is far less than that of the conventional single-map-type EKFS-LAM, while the computation time of the binary-tree-based method is shorter than that of the sequential method. It illustrates that the total computation time is largely reduced and the computational efficiency of the proposed algorithm is well optimized through the binary-tree-based strategy.

### 7.3. Victoria Park Dataset

**Figure 10.**
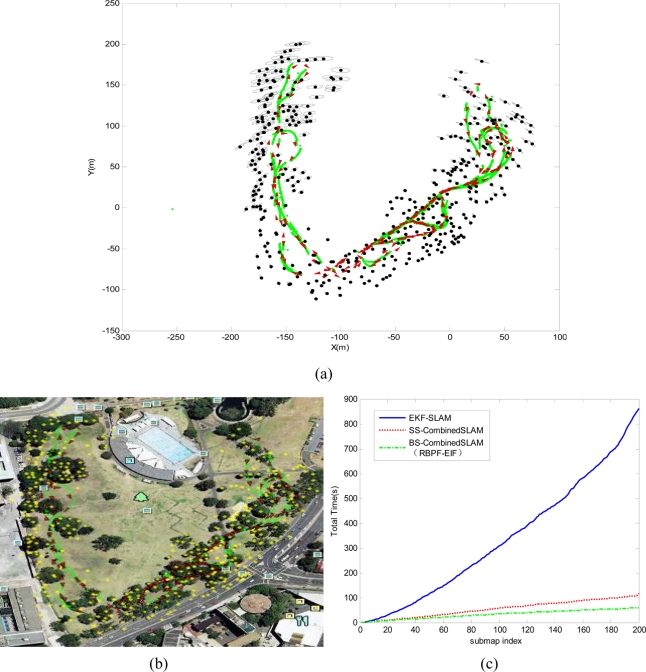
Results for the Victoria Park dataset. **(a)** Estimated map for the Victoria Park dataset (vehicle locations are drawn as red triangles, features are shown as black dots with ellipse covariance, and Green points are GPS readings). **(b)** Estimated results by the proposed algorithm were projected on Google Earth in order to compare the accuracy obtained (vehicle locations are drawn as red triangles, features are shown as yellow dots, and Green points are GPS readings). **(c)** Computational time of execution for the Victoria Park data: conventional single-map-type EKF-SLAM (blue), sequential submap-type Combined SLAM (red) and binary-tree-based submap-type Combined SLAM (green)

Finally, the Victoria Park dataset (available at http://www.mrpt.org/node/251) is employed to validate the proposed algorithm of this paper. Figure 10(a) shows the resulting global map composed of 200 local maps and the stored vehicle positions in the global state vector, where the black dots are position estimation of features with ellipse covariance, the vehicle locations in the global map are drawn as red triangles and the GPS readings are green points. The states recovered by our proposed algorithm are compared with the real-world environment of Victoria Park, as shown in Figure 10(b,c) that shows the total computational time of execution for the Victoria Park data. It can be seen clearly that the total cost of the proposed algorithm grows linearly and the total running time had been reduced dramatically when compared with EKFSLAM and sequential submap joining algorithm.

## 8. Conclusions

An efficient submap-based Combined SLAM algorithm for large-scale environments which combines the strengths of both RBPF-SLAM and EIF-SLAM had been studied in this paper. The RBPF-SLAM algorithm for local map building provides robust data association and improves the estimate validity, and the EIF-SLAM algorithm for submap joining globally allows uncertainty to be remembered over long robot trajectories. Simulation results clearly show that the proposed submap-based Combined SLAM algorithm has better consistency and accuracy in large-scale environments than the current typical algorithms. Furthermore, the proposed algorithm is still computationally efficient when the binary-tree-based submap joining strategy is introduced. The well known Victoria Park dataset has also been applied to verify the improved validity of the Combined SLAM approach and its computation time advantage.

## Figures and Tables

**Table 1.** The detailed CPU time by using algorithms of RBPF-EKF and RBPF-EIF.

**Algorithm**	**Submap joining**			
	**Data Association**	**Global map Update**	**Others**	**Total**
RBPF-EKF	4.4 s	1,445.1 s	3.4 s	1,452.9 s
RBPF-EIF	13.2 s	2.4 s	7.2 s	22.8 s
